# P-1167. Pharmacokinetics, Safety, and Tolerability of Funobactam in Combination with Imipenem/Cilastatin in Healthy Adult Elderly Male and Female Subjects

**DOI:** 10.1093/ofid/ofaf695.1360

**Published:** 2026-01-11

**Authors:** Jianguo Li, Qifeng Shi, Jing Feng, Meng Zhao, Hui Zhao, Anastasia McRoberts, Mitesh Sanghvi, Aramayis Kocharyan, Grigor Mamikonyan, Thomas C Marbury, Meijie Le

**Affiliations:** EVOPOINT BIOSCIENCES USA, INC, Chadds Ford, Pennsylvania; EVOPOINT BIOSCIENCES USA, INC, Chadds Ford, Pennsylvania; EVOPOINT BIOSCIENCES USA, INC, Chadds Ford, Pennsylvania; EVOPOINT BIOSCIENCES USA, INC, Chadds Ford, Pennsylvania; EVOPOINT BIOSCIENCES USA, INC, Chadds Ford, Pennsylvania; Pharmaron (Germantown) Lab Services Inc, Germantown, Maryland; Pharmaron (Germantown) Lab Services Inc, Germantown, Maryland; Clinartis, LLC, Hollywood, Florida; Clinartis, LLC, Hollywood, Florida; Orlando Clinical Research Center, Orlando, FL; EVOPOINT BIOSCIENCES USA, INC, Chadds Ford, Pennsylvania

## Abstract

**Background:**

Funobactam (formerly XNW4107) is a novel non-β-lactam diazabicyclooctane β-lactamase inhibitor with potent and selective direct activity against Ambler classes of A, C, D β–lactamases. Imipenem in combination with funobactam exhibits robust activity against carbapenem-resistant isolates of *Enterobacterales, P. aeruginosa,* and *A. baumannii*. This study evaluated pharmacokinetics (PK), safety, and tolerability of a single intravenous (IV) dose of imipenem 500 mg/ cilastatin 500 mg in combination with funobactam 250 mg in healthy adult elderly male and female subjects.

**Methods:**

Eight subjects with 6 and 2 subjects randomized to active treatment or placebo, respectively, were enrolled into each of following 3 study cohorts: Cohort 1: Healthy young females≥ 18 to ≤ 45 years; Cohort 2: Healthy elderly males ≥ 65 years and Cohort 3: Healthy elderly females ≥ 65 years. Blood and urine samples were collected to determine funobactam, imipenem and cilastatin concentrations by LC-MS/MS method. The safety and tolerability were assessed by monitoring adverse events, ECGs, vital sign measurements, physical examinations, and clinical laboratory data.

**Results:**

The mean (SD) of maximum concentration (C_max_), area under plasma concentration curve from zero to infinity (AUC_0-∞_,) plasma clearance (CL), volume of distribution at terminal phase (V_z_), half-life (t_1/2_) and renal clearance (CLr) for funobactam are shown in the table below:

**Table:** 

PKParameter (units)	Cohort 1 (N=6)	Cohort 2 (N=6)	Cohort 3 (N=6)
C_max_ (µg/mL)	16.3 (2.6)	19.2 (6.52)	18.2 (5.03)
AUC_0-inf_ (µg*h/mL)	45.0 (4.27)	68.2 (26.6)	63.0 (13.5)
CL (L/h)	5.59 (0.51)	4.08 (1.35)	4.11 (0.84)
V_Z_ (L)	20.2 (1.35)	21.3 (6.43)	18.6 (4.83)
t_1/2_ (h)	2.51 (0.11)	3.66 (0.39)	3.10 (0.46)
CLr	4.54 (1.47)	2.97 (0.63)	3.42 (0.73)

**Conclusion:**

Overall, there was no age and gender effect on the PK of funobactam after taking the difference in baseline creatinine into consideration. The single 60-minute IV administration of imipenem 500 mg/cilastatin500 mg in combination with funobactam 500 mg was safe and well tolerated in healthy adult elderly male and female subjects.

**Disclosures:**

All Authors: No reported disclosures

